# Methodological approaches to situational analysis in global mental health: a scoping review

**DOI:** 10.1017/gmh.2019.9

**Published:** 2019-06-13

**Authors:** J. K. Murphy, E. E. Michalak, H. Colquhoun, C. Woo, C. H. Ng, S. V. Parikh, L. Culpepper, C. S. Dewa, A. J. Greenshaw, Y. He, S. H. Kennedy, X.-M. Li, T. Liu, C. N. Soares, Z. Wang, Y. Xu, J. Chen, R. W. Lam

**Affiliations:** 1 Department of Psychiatry, University of British Columbia, Vancouver, British Columbia, Canada; 2 Department of Occupational Science and Occupational Therapy, University of Toronto, Toronto, Ontario, Canada; 3 Department of Psychiatry, University of Melbourne, Melbourne, Victoria, Australia; 4 Department of Psychiatry and Health Management & Policy, University of Michigan, Ann Arbor, Michigan, USA; 5 Department of Family Medicine, Boston University, Boston, Massachusetts, USA; 6 Department of Psychiatry and Behavioral Sciences, University of California Davis, Sacramento, California, USA; 7 Department of Psychiatry, University of Alberta, Alberta, Canada; 8 Shanghai CDC for Mental Health, Division of Training and Health Education, Shanghai, China; 9 Department of Psychiatry, University of Toronto, Toronto, Ontario, Canada; 10 Peking University, Institute of Population Research, Beijing, China; 11 Department of Psychiatry, Queen's University, Kingston, Ontario, Canada; 12 Hongkou District Mental Health Center, Shanghai, China; 13 Shanghai Mental Health Center, Shanghai Jiao Tong University School of Medicine

**Keywords:** Equity, formative research, global mental health, methods, situational analysis

## Abstract

Global inequity in access to and availability of essential mental health services is well recognized. The mental health treatment gap is approximately 50% in all countries, with up to 90% of people in the lowest-income countries lacking access to required mental health services. Increased investment in global mental health (GMH) has increased innovation in mental health service delivery in LMICs. Situational analyses in areas where mental health services and systems are poorly developed and resourced are essential when planning for research and implementation, however, little guidance is available to inform methodological approaches to conducting these types of studies. This scoping review provides an analysis of methodological approaches to situational analysis in GMH, including an assessment of the extent to which situational analyses include equity in study designs. It is intended as a resource that identifies current gaps and areas for future development in GMH. Formative research, including situational analysis, is an essential first step in conducting robust implementation research, an essential area of study in GMH that will help to promote improved availability of, access to and reach of mental health services for people living with mental illness in low- and middle-income countries (LMICs). While strong leadership in this field exists, there remain significant opportunities for enhanced research representing different LMICs and regions.

## Introduction

The global inequity in access to and availability of essential mental health services is well recognized and has been identified as a ‘grand challenge’ (Collins *et al*., 2011). The mental health treatment gap is approximately 50% in all countries, with up to 90% of people in the lowest-income countries lacking access to the mental health services they require (Saxena *et al*., 2007; Patel *et al*., 2010). Inequity is also found within countries; vulnerable groups, including people with low socioeconomic status (SES), women and sexual minorities, young people and people residing in rural areas are often most affected by mental health problems and are least likely to receive care (Saxena *et al*., 2007). The global mental health (GMH) treatment gap is indicative of a historic inequity in the prioritization and response to mental health compared with other health conditions (Votruba *et al*., 2016). The lack of essential treatment for many people suffering from mental illness in low- and middle-income countries (LMICs) has been described as a moral failure (Kleinman, 2009).

In the last decade, however, GMH has emerged as a response to the treatment gap in mental illness. Increased investment in GMH has led to the development and testing of innovative approaches to mental health service delivery in LMICs. In turn, as the evidence for effective interventions grows, so too does the need for a deeper understanding of how to implement and scale-up mental health services so that they effectively reach those in need (Eaton *et al*., 2011). Implementation science is a fundamental area of inquiry in GMH, representing a foundational component of sustained and effective interventions to diminish the treatment gap in LMICs (De Silva & Ryan, 2016).

Implementation research in GMH examines how to ensure that evidence-based practices are integrated into routine clinical care and throughout health systems, by ‘understand[ing] and address[ing] the behavioural, managerial, economic and social barriers’ to implementation and scale-up (De Silva & Ryan, 2016). Because it occurs within complex health systems, effective planning for implementation requires a deep understanding of context. Formative research, which ‘aims to determine how best to fit aspects of program design and/or implementation to the environmental and cultural context of its beneficiaries,’ (Bentley *et al*., 2014) is, therefore, a critical component of implementation research. Situational analysis, a type of formative research, is ‘an assessment of the current health situation and is fundamental to designing and updating national policies, strategies and plans’ (World Health Organization, 2018). Situational analysis is used to understand, from the outset of a study, the multiple interacting factors within a system that might affect implementation (Martin *et al*., 2016). These might include, for example, human and financial resources, health policies and plans, social determinants of health, and other interacting factors. Situational analyses in GMH, where mental health services and systems are poorly developed and resourced, are essential when planning for research and implementation. However, little guidance is available to inform methodological approaches to conducting these types of studies.

Equity in health, defined as ‘the absence of systematic disparities in health (or in the major social determinants of health) between groups with different levels of underlying social advantage/disadvantage,’ (Braveman & Gruskin, 2003) is at the core of GMH (Patel & Prince, 2010). The efforts of GMH to address inequity in mental health service delivery and access indicates a need for approaches to GMH implementation research that include equity as a key consideration (Rasanathan & Diaz, 2016). Explicitly integrating equity considerations into the design of situational analyses, therefore, could help to ensure that implementation planning includes strategies to promote equitable access to interventions by the most vulnerable and underserved populations.

We aimed to provide a scoping review of methodological approaches to situational analysis in GMH. We also assessed the extent to which GMH situational analyses include equity in their study designs. This review would serve as an important resource for GMH researchers in implementation research and help identify gaps and areas for future development in GMH.

## Methods

Scoping reviews are effective for summarizing and disseminating research findings and for identifying gaps in the literature (Arksey & O'Malley, 2005). They are particularly appropriate in fields with emergent findings and to ‘address questions beyond those related to intervention effectiveness’ (Levac *et al*., 2010). We used Arksey & O'Malley's (2005) methodological framework for scoping reviews to conduct the review and met the criteria of the PRISMA-Scoping Review (PRISMA-ScR) guidelines (Tricco *et al*., 2018). The objective of this review is to identify: (a) which methodological approaches for conducting situational analyses are commonly used in GMH; (b) the existing gaps and areas for further development in this area and, (c) to what extent, and how, equity considerations are captured in situational analyses in GMH.

We identified relevant studies through a search of PUBMED and PsychINFO databases using the search terms: Situational Analysis AND Mental Health AND Global, Situation Analysis AND Mental Health AND Global, and Formative Research AND Mental Health AND Global. The lead author (JM) initially conducted the database search in April and May of 2018. We also complemented our database search with a review of the reference lists of included articles to ensure we did not overlook relevant studies (Arksey & O'Malley, 2005). Based upon the broad definitions of situational analysis provided by the World Health Organization (WHO) (2018) and Martin *et al*. (2016), we sought to include papers that involved an assessment of contextual factors in the mental health system of LMIC or regions in advance of implementing mental health interventions, programs, plans and/ or policies.

**Fig. 1.**
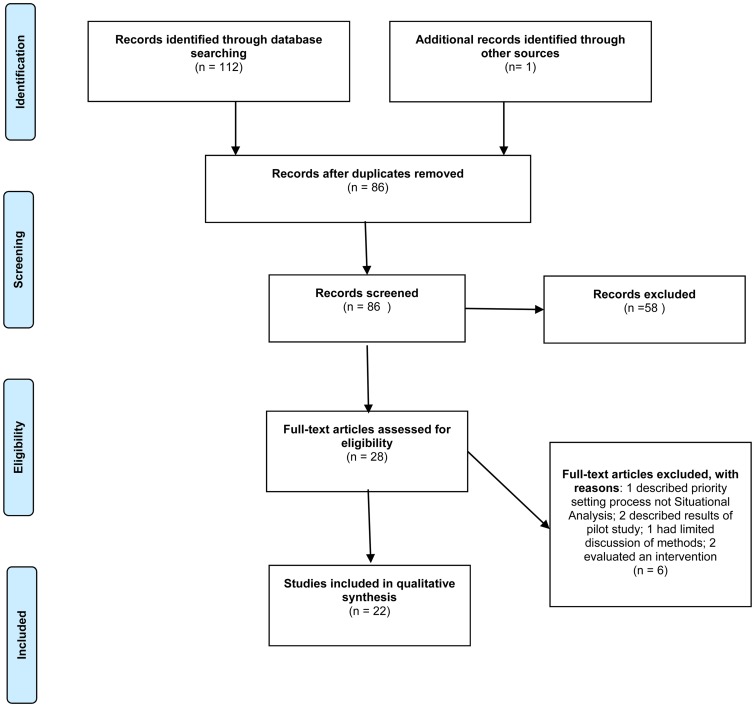
PRISMA diagram of scoping review literature search and selection.

Inclusion criteria were studies that: (1) took place in an LMIC context as defined by World Bank criteria (The World Bank, 2018), (2) described situational analysis methodology, defined as approaches that assessed the environmental and cultural context of health services delivery to inform the design and/or implementation of mental health interventions, programs or plans (Bentley *et al*., 2014; World Health Organization, 2018), and (3) focused on health systems and/ or service delivery research. We excluded studies that: (1) predominantly reported findings related to mental health epidemiology or intervention effectiveness or that did not otherwise constitute situational analyses or formative research (e.g. that took place after implementation, process evaluation, etc.), (2) were situated in high-income countries (HICs), and (3) were published in languages other than English. Figure 1 displays a PRISMA diagram of the search process. We began with an initial screening of the title and abstracts of records, followed by full-text screening. JM conducted the initial screening, which was then repeated and verified by co-author CW. Discrepancies were resolved by consensus with a third co-author (RWL).

We extracted and charted the results of the review using the following categories: study scope (e.g. national, regional or district level, single or multi-country), purpose, country of origin, objectives, methods, situational analysis tool characteristics and use, inclusion of equity in study design, and limitations as described by the study authors. The inclusion and exclusion criteria and the data extraction categories were decided through discussion amongst authors JM, EEM and RWL, with further consultation with the full study team. We analysed the results using a combination of numerical descriptive summary and qualitative thematic analysis (Levac *et al*., 2010). This allowed us to collate the results, which are summarized below according to the study review categories.

## Results

We identified 112 records through our database search and three additional records through a review of reference lists. A total of 27 duplicates were removed and another 58 were excluded after the title and abstract review. We excluded a further six records after reading the full text. A total of 24 papers were included in the review (see Fig. 1), representing 12 research programs s. Table 1 lists the studies that met inclusion criteria, the country or countries in which they took place, their objective(s), methods and limitations as identified by the study authors.

**Table 1.** Context and scope of included studies

Geographic region^a^	Number (*N* = 22)^b^	Percentage (%)
Sub-Saharan Africa	15	68.2
South Asia	9	40.9
East Asia and the Pacific	2	9.1
Latin America and the Caribbean	0	0
Middle East and North Africa	0	0
Scope		
Single country	8	36.4
Multi-country	14	63.6
National level	8	36.4
District/provincial level	12	54.5
Municipal level	2	9.1

a By World Bank region excluding North America which contains all High-Income Countries (Canada, USA, Bermuda).

b Numbers exceed total due to multi-country studies.

### Scope and context of studies

Table 2 displays the regional distribution and scope of the studies: 70.9% (*n*  =  17) of the 24 studies took place in Sub-Saharan Africa, 37.5% (*n*  =  9) took place is South Asia and 8.3% (*n*  =  2) two took place in East Asia. None were performed in Latin America, the Caribbean or Middle East and North Africa regions.

**Table 2.** Context and scope of included studies

Geographic region^a^	Number (*N* = 24)^b^	Percentage (%)
Sub-Saharan Africa	17	70.9
South Asia	9	37.5
East Asia and the Pacific	2	8.3
Latin America and the Caribbean	0	0
Middle East and North Africa	0	0
Scope		
Single country	10	41.6
Multi-country	14	58.3
National level	10	41.6
District/provincial/state level	12	50.0
Municipal level	2	8.3

a By World Bank region excluding North America which contains all High-Income Countries (Canada, USA, Bermuda).

b Numbers exceed total due to multi-country studies.

For this analysis, studies are counted as ‘single country’ if they reported results from only one country in a paper, regardless of their broader affiliation with multi-country research programs. Of the studies, 14 (58.3%) took place in multiple countries, with high representation in this review of papers published by consortia such as the Programme to Improve Mental Health Care (PRIME) (Jordans *et al*., 2013; Hanlon *et al*., 2014; Asher *et al*., 2015; Luitel *et al*., 2015; Shidhaye *et al*., 2015; Baron *et al*., 2016; Hailemariam *et al*., 2016; Kigozi *et al*., 2016; Angdembe *et al*., 2017) and the Emerging Mental Health Systems in LMICs (EMERALD) study (Abdulmalik *et al*., 2016; Upadhaya *et al*., 2016; Mugisha *et al*., 2017). Ten (41.6%) focused on a single country. Twelve (50.0%) of the studies focused on the district, provincial or state level, while 10 (41.6%) focused at the national level, and two (8.3%) took place at a municipal level.

### Objectives of included studies

The broad objectives of the included studies are categorized by theme in Table 3. Twelve (50%) of the studies conducted situational analyses in advance of developing mental health policies or plans at national or regional levels. Of the 10 countries with the primary goal of informing mental health policy and planning, six were part of the PRIME study (Jordans *et al*., 2013; Hanlon *et al*., 2014; Luitel *et al*., 2015; Shidhaye *et al*., 2015; Kigozi *et al*., 2016; Petersen *et al*., 2016), which aims to develop, test and scale-up mental health plans in selected districts of each of the study's participating countries (Ethiopia, India, Nepal, South Africa, and Uganda) (Lund *et al*., 2012). Studies targeting policy and planning used situational analyses to understand the current state of mental health governance (Abdulmalik *et al*., 2016), to assess mental health resources (financial, human resource, infrastructure), to identify current practice and gaps in mental health service delivery, and to understand the broader health and socioeconomic context of the countries or regions of interest (Ofori-Atta *et al*., 2010; Sikwese *et al*., 2010; Jordans *et al*., 2013; Esan *et al*., 2014; Hanlon *et al*., 2014; Luitel *et al*., 2015; Baron *et al*., 2016; Kigozi *et al*., 2016; Petersen *et al*., 2016; Upadhaya *et al*., 2016; Olofsson *et al*., 2018).

**Table 3.** Study objectives

Study purpose	Number (*N* = 24)	Percentage (%)
Mh policy and plan development	12	50.0
Development or design of mh programmes or services	7	29.2
Development or design of specific interventions	5	20.8

Seven (29.2%) conducted situational analyses and formative research to inform the development or design of mental health programmes or services. Objectives included the development of a package of community-based mental health and mobile health services (Angdembe *et al*., 2017), supporting the integration of mental health services into primary health care (Bhana *et al*., 2010; Baron *et al*., 2016; De Kock & Pillay, 2016; Mugisha *et al*., 2017) and improving the availability and accessibility of treatment for specific mental health conditions (Hailemariam *et al*., 2016; Tekola *et al*., 2016).

Finally, five (20.8%) papers used situational analyses and formative research before developing specific clinical mental health interventions. These sought to: inform the design of acceptable and feasible interventions for the study context (Asher *et al*., 2015; Davies *et al*., 2016; Maulik *et al*., 2016; Yu *et al*., 2017), assess contextual and implementation factors prior to pilot testing of interventions (Dos Santos & Wolvaardt, 2016), understand the sociocultural context of intervention delivery, including explanatory models of mental illness and stigma (Davies *et al*., 2016; Maulik *et al*., 2016; Yu *et al*., 2017) and to identify factors that might affect the scale-up and sustainability of interventions (Asher *et al*., 2015; Dos Santos & Wolvaardt, 2016).

### Study methodologies

Methodological approaches used for situational analyses can be categorized into three themes: situational analyses using secondary data review, qualitative research and priority setting exercises. Table 4 displays the methodological approaches and the number of methods used by each study. Fourteen (58.3%) of the individual studies used one method, six (25.0%) used two and two (8.3%) combined three methodological approaches. Because many of the papers represent the PRIME and EMERALD research consortia, we also note studies that are associated with them and collated approaches used by each programme.

**Table 4.** Methodological approaches by study

Individual study	Secondary data analysis using tool	Secondary data analysis not using tool	Qualitative methods	Priority setting exercises
Abdulmalik *et al.* (2016) [E]	X			
Angdembe *et al.* (2017) [P]			X	
Asher *et al.* (2015) [P]	X		X	X
Baron *et al.* (2016) [P]	X			
Bhana *et al.* (2010)	X		X	
Davies *et al.* (2016)			X	
De Kock and Pillay (2016)		X	X	
Dos Santos and Wolvaardt (2016)			x	
Esan *et al.* (2014)	X			
Hailemariam *et al.* (2016) [P]			X	
Hanlon *et al.* (2014) [P]	X			
Jordans *et al.* (2013) [P]			X	X
Kigozi *et al.* (2016) [P]			X	
Luitel *et al.* (2015) [P]	X			
Maulik *et al.* (2016)			X	
Mugisha *et al.* (2017) [E]	X			
Ofori-Atta *et al.* (2010)	X		X	
Olofsson *et al.* (2018)	X		X	
Petersen *et al.* (2016)	X		X	X
Sikwese *et al.* (2010)	X		X	
Shidhaye *et al.* (2015) [P]	X		X	
Tekola *et al.* (2016)		X	X	X
Upadhaya *et al.* (2016) [E]	X			
Yu *et al.* (2017)			X	
PRIME study [P]	X		X	X
EMERALD study [E]	X			

Seventeen (70.8%) of studies included qualitative research, using key informant and in-depth interviews and/or focus group discussions (Bhana *et al*., 2010; Ofori-Atta *et al*., 2010; Sikwese *et al*., 2010; Jordans *et al*., 2013; Asher *et al*., 2015; Shidhaye *et al*., 2015; Davies *et al*., 2016; De Kock & Pillay, 2016; Dos Santos & Wolvaardt, 2016; Hailemariam *et al*., 2016; Kigozi *et al*., 2016; Maulik *et al*., 2016; Petersen *et al*., 2016; Tekola *et al*., 2016; Angdembe *et al*., 2017; Yu *et al*., 2017; Olofsson *et al*., 2018). Qualitative studies involved a range of participants, including: policymakers, mental health specialists, primary care providers, other healthcare providers (e.g. maternal health, HIV), community health workers, traditional healers, lay providers, service users, families and caregivers, police officers, teachers, community members and mental health experts. Many of the qualitative studies sought to understand existing mental health service availability in the study context, with qualitative methods providing a deeper understanding of current mental health practice, needs and gaps in service, and barriers to implementing policy, plans or interventions (Bhana *et al*., 2010; Ofori-Atta *et al*., 2010; Sikwese *et al*., 2010; Shidhaye *et al*., 2015; De Kock & Pillay, 2016; Dos Santos & Wolvaardt, 2016; Hailemariam *et al*., 2016; Kigozi *et al*., 2016; Maulik *et al*., 2016; Tekola *et al*., 2016; Olofsson *et al*., 2018). Studies also used qualitative methods to understand acceptability, including the acceptability of integrating mental health care into existing services (e.g. primary care, HIV service delivery, community-based care) (Jordans *et al*., 2013; Dos Santos & Wolvaardt, 2016; Petersen *et al*., 2016; Angdembe *et al*., 2017), the acceptability of treatment options and interventions (Asher *et al*., 2015; Yu *et al*., 2017) and gaps in human resources for mental health (Sikwese *et al*., 2010).

Sixteen (66.6%) of the studies reviewed secondary data; 14 used a specifically designed situational analysis tool and two studies did not use a tool. Tools used for situational analysis varied, with the majority (7) using the PRIME situational analysis tool, which was specifically designed for the PRIME study (Hanlon *et al*., 2014; Asher *et al*., 2015; Luitel *et al*., 2015; Shidhaye *et al*., 2015; Baron *et al*., 2016; Petersen *et al*., 2016; Olofsson *et al*., 2018). Of these, one was not part of the PRIME consortium (Olofsson *et al*., 2018). In this case, the investigators adapted the PRIME tool for length and for relevance to children's mental health in the Cambodian context. Two additional teams developed their own situational analysis frameworks (Esan *et al*., 2014; Upadhaya *et al*., 2016), while others used or adapted the WHO-Assessment Instrument for Mental Health (AIMS) tool, and WHO checklists for mental health policy, plans and legislation (Bhana *et al*., 2010; Ofori-Atta *et al*., 2010; Sikwese *et al*., 2010; Abdulmalik *et al*., 2016). Details of the PRIME Situational Analysis Tool, the WHO-AIMS and the tools developed by Esan *et al*. (2014) and Upadhaya *et al*. (2016) are provided in Table 5. Two studies (De Kock & Pillay, 2016; Tekola *et al*. 2016) did not use tools. All studies using secondary data review methodology reviewed documents that were available in the public domain, including health surveillance and census data, mental health legislation, policy and plans, academic publications, government reports from mental health, general health and related sectors, media reports and medical records. In the majority of studies, data were collected by members of study research teams and often supplemented with personal communication with local mental health experts and stakeholders. Sikwese *et al*. (2010) and Ofori-Atta *et al*. (2010) involved key stakeholders in the completion of the WHOAIMS.

**Table 5.** Characteristics of situational analysis tools and measures

Tool	Primary reference	Studies using tool	Description	Key components
Prime situational analysis tool	Hanlon *et al*. (2014)	Asher *et al*. (2015) Baron *et al* (2016) Kigozi *et al*. (2016) Luitel *et al*. (2015) Olofsson *et al*. (2018) Petersen *et al*. (2016)	Developed for the PRIME study to enable use of secondary data and applicability to smaller (e.g. district) population units. Designed to assess necessary implementation factors for mhGAP Implementation Guide (WHO, 2010) in primary care settings.	1: Relevant context 2: Mental health politics, policies and plans 3: Mental health treatment coverage 4: District level health services 5: Community 6: Monitoring and evaluation
WHO assessment instrument for mental health systems (WHO-AIMS) VERSION 2.2	World Health Organization. Assessment instrument for mental health systems. (2015) Geneva: WHO-AIMS.	Bhana *et al*. (2010) Mugisha *et al*. (2017)	Developed by WHO in 2005 for the collection of information on mental health systems in a country or region with the goal of improving mental health systems.	1: Policy and Legislative Framework 2: Mental Health Services 3: Mental Health in Primary Health Care 4: Human Resources 5: Public Education and Links with Other Sectors 6: Monitoring and Research
Situational analysis tool to assess mental health care in anglophone west Africa	Esan *et al*. (2014)	Esan *et al*. (2014)	Developed both to assess mental health situation in countries to inform planning processes and to capture change over time. In addition to more standardized measures it includes qualitative component to understand cultural factors, historical context, etc.	1.Mental health service availability 2. Mental health policy 3. Prevalence of mental health conditions 4. Legislation for mental health human rights 5. Mental health financing 6. Human resources for mental health availability 7. Mental health specialist services needs 8. Availability of medications 9. Organizations working in mental health
HMIS situation analysis tool for data collection	Upadhaya *et al*. (2016)	Upadhaya *et al*. (2016)	Developed by study authors to assess health management information systems in EMERALD study countries	1. Background of HMIS 2. HMIS plans and policies 3. Data recording and collating processes 4. Monitoring, evaluation and feedback procedures 5. Dissemination and use of data 6. Human resources 7. Availability of mental health indicators 8. Coordination and linkages 9. Open section for other relevant information

Four (16.7%) of the studies used priority setting exercises as part of their situational analyses and formative research. Three of these studies (Jordans *et al*., 2013; Asher *et al*., 2015; Petersen *et al*., 2016) used Theory of Change workshops, in which stakeholders are convened to ‘map a causal chain of pre-conditions (or preliminary outcomes), assumptions and interventions leading to an ultimate outcome’ (Jordans *et al*., 2013 3). Theory of Change methodology is used by the PRIME program to map pathways to developing mental health plans and to encourage stakeholder engagement (Breuer *et al*., 2014). Tekola *et al*. (2016) also used workshops for priority setting but did not use the Theory of Change methodology.

The priority-setting workshops were used to inform the development of an intervention (Asher *et al*., 2015) and mental health plans (Jordans *et al*., 2013; Baron *et al*., 2016) and to capture stakeholder perspectives on the mental health situation, and challenges and opportunities for service improvement (Tekola *et al*., 2016). Participants included community leaders, service providers and managers, policy makers, end users, families, mental health experts and representatives of local and international non-governmental organizations.

### Equity considerations in study design

Three studies explicitly included equity considerations in their study designs (Abdulmalik *et al*., 2016; Hailemariam *et al*., 2016; Mugisha *et al*., 2017). Abdulmalik *et al*. (2016) used a framework (Siddiqi *et al*., 2009) to guide the design of the qualitative component of their study that included an ‘ethics and inclusiveness’ component. They identified multiple factors related to equity in their study design and analysis, including stigma and discrimination, low prioritization of mental health, low mental health awareness by policymakers, financial coverage available to people accessing mental health care (e.g. public insurance coverage), and rights protection for people with mental health disorders.

The overall goal of Hailemariam *et al*.,'s (2016) study was to ‘inform the development of equitable and accessible mental healthcare integration into primary mental health services for people with severe mental disorders in Ethiopia. That qualitative study identified barriers to equitable access to services, defining equitable access in terms of the possible effect of gender, physical disability, socioeconomic status and location of the residence on access to mental health care.

Mugisha *et al*.,'s (2017) situational analysis of the health system context for primary care integration across the EMERALD study countries (Ethiopia, India, Nepal, Nigeria, South Africa and Uganda) included the analysis of ‘issues of equity in relation to existing policies’ in the study design. This included examining if and how considerations of gender, poverty, disability and other vulnerable populations are considered in mental health policy and provision.

Five other studies implicitly addressed issues related to equity but did not incorporate it in their study design; that was addressed by discussing the impact of poverty on help-seeking (Angdembe *et al*., 2017), the relationship between poverty and mental health and gaps in social protection for people with mental illness (Ofori-Atta *et al*., 2010), the implications of community mobilization for social inclusion, reduced stigma and improving economic status (Asher *et al*., 2015), the impact of poverty and living circumstances on women's mental health (Davies *et al*., 2016) and the role of mental health information systems in capturing equity in service distribution for women, people with low socioeconomic status, and rural inhabitants (Shidhaye *et al*., 2015).

### Identified limitations

Seven studies did not describe limitations related to situational analysis methodology (Ofori-Atta *et al*., 2010; Sikwese *et al*., 2010; Esan *et al*., 2014; Abdulmalik *et al*., 2016; Dos Santos & Wolvaardt, 2016; Kigozi *et al*., 2016; Petersen *et al*., 2016).

The most common limitation identified by those that did describe limitations of situational analyses related to use of secondary data (Hanlon *et al*., 2014; Luitel *et al*., 2015; Shidhaye *et al*., 2015; Baron *et al*., 2016; De Kock & Pillay, 2016; Upadhaya *et al*., 2016; Mugisha *et al*., 2017). Many countries have mental health information systems that are developing, resulting in limited availability of documents related to the specific mental health contexts, or documents that were not up to date. Studies that described this limitation noted that they attempted to mitigate this issue, however, by supplementation with personal communications with local mental health stakeholders.

Several studies without a qualitative component identified an inability to include findings on knowledge, attitudes and beliefs of stakeholders as a study limitation. Some authors indicated that complementary qualitative studies were being or would be untaken to capture this information (Hanlon *et al*., 2014; Luitel *et al*., 2015; Upadhaya *et al*., 2016) and one cited lack of resources to include a qualitative component in the study (Mugisha *et al*., 2017).

The most commonly cited limitation of qualitative studies was the limited scope of the study area and of study participants, which could limit generalizability of findings (Jordans *et al*., 2013; De Kock & Pillay, 2016; Angdembe *et al*., 2017; Yu *et al*., 2017). Other limitations included language barriers between researchers and study participants, requiring the translation of interview transcripts and the risk of loss of meaning or misunderstanding of findings (Angdembe *et al*., 2017; Olofsson *et al*., 2018).

For studies using priority-setting exercises, limitations included the risk of social desirability bias due to the study's affiliation with the national government (Asher *et al*., 2015). The type of participants included, the use of purposive sampling and the priorities of participants were also acknowledged as potential limitations. Jordans *et al.* (2013) described a predominance of mental health experts from urban settings as a potential limitation. They also acknowledged that the priority setting outcomes themselves may have been biased by the experience and knowledge of participants; they were surprised to find that developmental disorders were given low priority and noted that this may have been due to the shortage of children's mental health experts in the country. Tekola *et al*. (2016) cite the lack of traditional healers as participants as a limitation given their important role in the Ethiopian context.

## Discussion

This review identified a number of themes that are important to implementation research in GMH, pointing both to gaps and strengths in the field. Improving GMH depends not only on implementing evidence-based interventions, but also on modifying and implementing interventions that anticipate local implementation barriers across behavioural, managerial, economic, and social domains. Understanding these complex barriers, and ensuring that equity in health can be championed, requires sophistication in the situational analysis. Our review explored various methodological approaches to situational analysis as a guide to enhancing implementation strategies. We discovered that relatively few studies address situational analyses in GMH, with additional key findings noting the paucity of local mental health system data and an absence of consideration of equity. Fortunately, our review also identified the development of several situational analysis tools that can serve as key resources, and a methodological strategy—mixed methods—that can address challenges.

We found that the scope of published situational analyses and formative research in GMH is limited. Twelve of the studies included in the review were representative of the PRIME or EMERALD studies, which both include sites in Ethiopia, India, Nepal, South Africa and Uganda, while EMERALD also includes Nigeria (Lund *et al*., 2012; Mugisha *et al*., 2017). PRIME and EMERALD are providing leadership in GMH implementation research (De Silva & Ryan, 2016) and currently make up the majority of the published literature in this nascent field. There is a gap, however, in research representing Latin America and the Caribbean and the Middle East and North Africa, and there is limited research from the East Asia and Pacific region. There is a need for expanded investment in and dissemination of situational analyses from other geographic regions and other programs. The importance of formative research has been recognized in other global health disciplines (Scott *et al*., 2018), but neither methodological approaches to formative research nor its importance is yet sufficiently described in the literature (Bentley *et al*., 2014).

Though outside the scope of this review, an informal search of situational analysis literature and tools from HICs also revealed a gap. There appears to be limited peer-reviewed literature detailing situational analysis tools and methods in mental health or the broader health sector in HICs, though some examples are found in the grey literature. For example, Alberta Health Services in the Canadian province of Alberta conducted a situational analysis of the use of impact assessments in the health sector (Alberta Health Services, 2017). The methodology for this situational analysis was informed by a planning and evaluation framework that was developed internally and was not extensively described in the report. Situational analyses using methods such as Strengths, Weaknesses, Opportunities and Threats (SWOT) analysis seem to predominantly appear in the business and marketing domain in HICs. This dearth of literature on situational analyses from both LMICs and HICs represents a considerable opportunity for GMH researchers to provide leadership in this area and to make a substantial contribution to implementation research in mental health and in global health more broadly.

Many of the studies included in this review used or developed situational analysis tools that provide a comprehensive and systematic template for the analysis of complex factors at multiple levels of a mental health system. The PRIME investigators developed their own tool, adapted from WHO-AIMS, to assess district-level mental health needs (Hanlon *et al*., 2014). Esan *et al*. (2014) developed a tool to assess mental health systems across Anglophone West African countries and included qualitative components to capture the cultural and historical context of mental health. These tools represent an important foundational resource that can be used by GMH researchers conducting situational analyses and can be adapted to meet the needs of specific contexts and study objectives (Olofsson *et al*., 2018).

While the tools represent a considerable asset to GMH researchers, their potential to contribute to robust situational analysis research relies on the quality of their implementation and on the availability of secondary data. A major limitation identified by study authors was the often limited availability of mental health system data. Mental health information systems in LMICs are at varying stages of development and may be disjointed and under-resourced. Their strengthening has been identified as a priority in GMH (Ryan *et al*., 2015). Publicly available mental health data are essential for completing comprehensive situational analyses, but the limited or inconsistent availability of data will remain a considerable limitation as long as mental health systems are still advancing. Further, most situational analysis tools do not capture the knowledge, beliefs and attitudes of mental health system stakeholders, which are essential to understanding culture, context and potential barriers and drivers of implementation (Proctor *et al*., 2009; Aarons *et al*., 2011).

Using mixed methods to conduct situational analyses could help to mitigate some of these challenges, along with the limitations of both qualitative research and priority setting exercises described above. Mixed methods approaches combine both quantitative and qualitative data collection, analysis and interpretation in order to provide a deeper understanding of phenomena (Johnson *et al*., 2007; Creswell & Clark, 2011; Ostlund *et al*., 2011). Mixed methods approaches are effective for triangulating data, or using various data sources or methods to improve depth of understanding and validate research findings (Carter *et al*., 2014) and for capturing the complexity of health systems (Tariq & Woodman, 2013). Despite the potential utility of mixed methods research, only six of the included studies used more than one method. The PRIME study was represented by nine studies in this review and while not all studies used mixed methods, the combined studies did involve mixed methods and priority setting exercises. While mixed methods can be more resource intensive, they should be considered when conducting situational analyses in order to ensure a more robust and comprehensive analysis of mental health systems. Mixed methods studies should be encouraged and supported by GMH implementation funding agencies to promote rigorous implementation research from the inception of research studies to their conclusion.

Finally, only three of the 24 studies explicitly considered equity in their study design. While the PRIME situational analysis tool does include indicators related to equity, these were not explicitly discussed in the papers included in this review. Equity on a global scale is a fundamental concern of GMH, which aims to close the gap in treatment within and between countries, improving access to and availability of treatment and improving the lives of people suffering from mental disorders (Patel *et al*., 2011). Implementation science has the potential to improve equity in health service delivery (Rasanathan & Diaz, 2016). Implementation research in GMH, beginning with situational analyses, can provide important insight into equity gaps and opportunities to improve mental health equity. Including frameworks for assessing equity considerations in situational analysis, including mixed methods approaches that help to mitigate challenges related to poor data availability, study designs would allow for equity to be considered in the design, implementation and scale-up of interventions, programs and policies and for equity outcomes to subsequently be monitored. Equity is a broad and complex concept, the dimensions of which should be explored in further depth in relation to GMH implementation.

## Limitations

We only included English language studies in this review which may have led to the omission of relevant research in this area published in other languages. It is also likely that situational analyses and formative studies have been conducted in GMH using different terminology and were thus not identified with the search terms used in this scoping review. The use of MeSH terms in the search may also have yielded additional results. We are confident, however, that the review has captured a representative sample of the GMH literature on situational analysis methodology in GMH, and that it offers a useful overview of current practice and guidance on next steps in this important field of research.

## Conclusions

Implementation research is an essential area of study in GMH and will help to promote improved availability of, access to and reach of mental health services for people living with mental illness in LMICs. Formative research, including situational analysis, is an essential first step in conducting robust implementation research. While strong leadership in this field exists, there remain significant opportunities for enhanced research representing different LMICs and regions.

This review of existing methodological approaches in situational analysis for GMH reveals limitations and opportunities that can inform the design of future studies. The field will benefit from prioritizing mixed methods approaches to implementation research and including equity considerations in the study design of formative research, including situational analyses.
